# SIX3 as a Regulator of Development and Disease

**DOI:** 10.3390/jdb14010013

**Published:** 2026-03-06

**Authors:** Ana Beatriz Matos, Laura Jesus Castro, Torcato Martins

**Affiliations:** Department of Medical Sciences, Institute of Biomedicine, University of Aveiro, Agra do Crasto, 3810-193 Aveiro, Portugal; anabmatos@ua.pt (A.B.M.); lauracastro@ua.pt (L.J.C.)

**Keywords:** Optix/SIX3, SIX transcription factors, neuronal development and homeostasis, transcriptional regulation, type-II diabetes, oncogenesis

## Abstract

Transcriptional regulation is pivotal for developmental processes and cell fate specification in homeostasis. One particularly relevant group of transcription factors is the sine oculis homeobox (SIX) family, which is involved in a wide range of molecular processes from development to tissue maintenance. Within this family, distinct subfamilies exhibit specific DNA-binding preferences and can function as transcriptional activators or repressors. In this review, we focus on the Optix/SIX3–SIX6 subfamily and discuss their roles as transcriptional regulators, as well as the consequences of their deregulation for neuronal and ocular development and for the maintenance of tissue homeostasis. We further examine how SIX3 can act either as a tumour suppressor or as a marker of poor prognosis in different cancer types. Moreover, we summarize recent findings on the role of SIX3 in pancreatic β cells and highlight emerging evidence that SIX2 also contributes to β-cell identity and regulatory stability. Downregulation of SIX2 and SIX3 alters gene regulatory programs associated with β-cell homeostasis and contributes to type 2 diabetes. As accumulating evidence links members of the SIX family to cancer and metabolic disease, it is crucial to characterize how these transcription factors regulate cell identity, with important implications for disease mechanisms and therapeutic strategies.

## 1. Introduction

Embryonic development and the maintenance of cellular identity depend on finely regulated gene expression programs that coordinate fundamental processes such as cell proliferation, differentiation, and tissue patterning. These programs are largely controlled by transcription factors, which integrate positional information and signals from multiple signalling pathways to activate or repress specific sets of target genes [1,2]. Dysregulation of these mechanisms can compromise normal development and contribute to the onset of pathologies, including congenital defects and cancer [3].

Among transcription factors with central roles in developmental control are the homeobox proteins, a highly conserved group that functions within regulatory networks responsible for cellular specification and embryonic pattern formation [4,5]. These proteins often act as regulatory hubs, linking extracellular signalling pathways to specific transcriptional outputs, thereby ensuring the temporal and spatial coordination of developmental processes [6,7]. Within this broad class, the sine oculis homeobox (SIX) family represents a functionally diverse group of transcription factors that exemplifies how subtle structural differences can generate distinct regulatory outputs during development.

**Figure 1:**
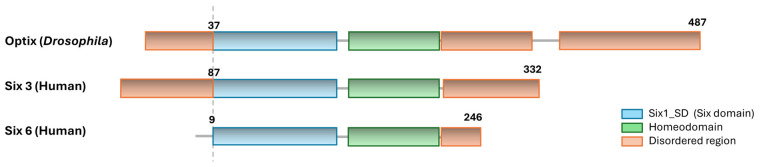
Structural organization of Optix, SIX3, and SIX6 proteins. Schematic representation of the protein architecture of Optix, SIX3 and SIX6. The conserved SIX domain (blue) mediates interactions with co-factors such as Groucho/TLE and AES, while the homeodomain (green) is responsible for DNA binding. Disordered regions (orange) potentially mediate species-specific interactions and regulatory versatility. Amino acid positions for start of the Six Domain and the size of each protein are indicated.

The SIX family constitutes a group of developmental regulators that are essential for the establishment and patterning of anterior structures throughout metazoan evolution [8,9]. Based on sequence similarity and DNA-binding specificity, SIX proteins are classically divided into three major subfamilies, SIX1/SIX2, SIX3/SIX6, and SIX4/SIX5, which exhibit both overlapping and distinct biological functions [9,10]. SIX proteins share a conserved molecular architecture composed of two main functional domains: the SIX domain, which mediates protein–protein interactions, and the homeodomain, responsible for sequence-specific DNA binding [8,11] (Figure 1). Although these domains are highly conserved, relatively minor variations within the homeodomain across SIX subfamilies result in distinct DNA-binding preferences, enabling differential recognition of cis-regulatory motifs and contributing to functional diversification [8,12].

While the conserved SIX and homeobox domains define the core DNA-binding and interaction properties of SIX proteins, additional regions contribute to their regulatory versatility. SIX family proteins contain poorly conserved N-terminal and, in particular, C-terminal regions that display features of intrinsically disordered regions (IDRs) [13,14]. These flexible regions, which are common in transcription factors, lack stable tertiary structure but play important functional roles by facilitating dynamic and context-dependent interactions with cofactors and regulatory complexes, thereby fine-tuning transcriptional activity across developmental and homeostatic contexts [13,15,16].

Interestingly, a minor change in the homeodomain between subfamilies makes them recognize different DNA sequences. While the subfamilies SIX1/SIX2 and SIX4/5 preferentially bind the TCAGGTTTC nucleotide motif, an almost identical homeodomain in the SIX3/SIX6 family binds conserved ATTA/TAAT nucleotide motifs within regulatory regions of its target genes [17,18,19]. Thus, it is not surprising that other SIX family members present some redundancy and often co-regulate the same promoters, for example, SIX1, SIX2, SIX4, and SIX5 can bind MEF3 or ARE elements in genes such as *aldolase A* [18], *myogenin* [18,20], or the *Na*, *K-ATPase α1 subunit* [17], whereas SIX3 does not. On the other hand, the SIX3/SIX6 subfamily, besides having a similar structure, regulates different targets and is involved in distinct functions [21].

In this review, we are going to explore the uniqueness of SIX3, where its structural organization enables it to function as a transcriptional activator or repressor according to the developmental context [22,23,24]. Moreover, we will explore how Optix/SIX3 is regulated both transcriptionally and by post-translational modifications and the impact on neuronal development and tissue homeostasis.

## 2. Regulation of SIX3 Function

SIX3 is a highly conserved transcription factor and a central member of the SIX family, a group of developmental regulators essential for the establishment and patterning of anterior structures throughout metazoan evolution. Thus, due to its essential roles, SIX3 is regulated at multiple levels (Table 1).

At the transcriptional level, pioneer studies come from the analysis of the PAX6/Ey transcription factor targets [25]. Genome-wide binding analyses have identified *optix* as a direct transcriptional target of Ey, the Pax6 homolog and master regulator of retinal determination in *Drosophila*. Direct Ey occupancy at *optix* regulatory regions places *optix* downstream of *ey* within the retinal determination gene network, highlighting its early integration into the genetic hierarchy controlling eye field specification [25].

Subsequent work in other model organisms has expanded the repertoire of transcriptional regulators of SIX3/SIX3.1 in vertebrates. In zebrafish, *six3.1* expression is directly enhanced by Pax6.1, which binds to regulatory elements required for retinal expression and increases transcriptional activity in co-injection assays, supporting a conserved regulatory interaction between Pax6 and SIX3 during eye development in vertebrates [26]. In medaka fish, systematic trans-regulatory screening has confirmed Pax6 as a direct regulator of *six3.2* and also identified additional factors such as Msx2, Pbx1, and Tcf3 (Table 1) as bona fide direct regulators of distinct spatial–temporal domains of *six3.2* expression in the developing forebrain, indicating that SIX3 integrates inputs from multiple upstream transcription factors in vertebrate neurodevelopment [27].

In addition to classical transcription factors, SIX3 transcription is modulated by post-transcriptional and chromatin-associated regulators. The microRNA miR-4306 directly targets the 3′UTR of SIX3 mRNA, reducing its expression and inhibiting proliferation, invasion, and EMT in esophageal squamous cell carcinoma cells, while low miR-4306 levels are associated with more aggressive tumour phenotypes [28]. In addition, long noncoding RNAs such as Six3OS1 also modulate SIX3 activity by interacting with transcriptional co-regulators and histone modification enzymes, adding another layer of transcriptional regulation for this gene during retinal development [29]. Furthermore, the co-regulator MTA1 interacts with the SIX3 promoter and recruits histone deacetylase and chromatin remodelling complexes to repress SIX3 transcription. Thus, loss of MTA1 increases SIX3 mRNA and protein levels, identifying MTA1 as a key transcriptional modulator of SIX3 [30].

Interestingly, studies in zebrafish also show that SIX3 binds directly to its own promoter, suggesting a potential autoregulatory mechanism that may help sustain appropriate SIX3 expression levels during early neural and eye field development, although whether this behaviour is tissue or species-specific requires further investigation.

From an evolutionary perspective, the *Drosophila* gene *optix* is the single member of this subfamily and gene duplication events within the SIX family gave rise, in vertebrates, to SIX3 and SIX6, two closely related transcription factors conserved across species and present in diverse model organisms, including *Xenopus laevis*, *Danio rerio*, and *Mus musculus* [31,32]. Consistent with this evolutionary relationship, Optix, the functional ortholog of SIX3/SIX6 in *Drosophila melanogaster*, encodes a SIX-class homeodomain transcription factor containing both a conserved SIX domain and a homeobox domain (Figure 1) [21]. Despite this conserved core architecture, the N- and C-terminal regions outside these structured domains are highly divergent, which might contribute to species-specific protein–protein interactions and regulatory functions, suggesting that these less conserved regions underlie adaptations of SIX3/Optix-controlled transcriptional programs in distinct developmental contexts [33,34].

Depending on the cellular context and recruited cofactors, SIX3 can function as both a transcriptional activator and repressor, participating in regulatory networks finely tuned to embryonic developmental requirements [7,31]. Historically, Rhodopsin was the first directly validated binding target of SIX3 [35], but accumulating evidence has shown other genes directly activated by SIX3 (Table 2). One example is the binding of SIX3 to the Foxg1 promoter, where it binds a conserved enhancer element and increases luciferase reporter activity in a dosage-dependent manner, indicating a direct activator function in neural development [36,37]. In addition, SIX3 acts as a direct activator of key regulators of neural and ocular identity, including Pax6 and Sox2 (Table 2).

**Figure 2:**
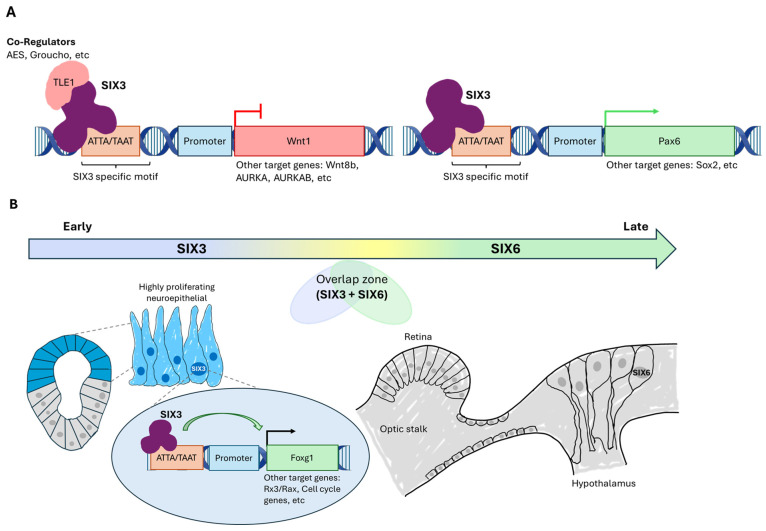
Functional transition from SIX3 to SIX6 in the regulation of signalling pathways and progenitor maintenance during forebrain and eye development. (**A**) SIX3 binds ATTA/TAAT-containing motifs and recruits co-repressors through its SIX domain (TLE, AES, WDR domains), leading to repression of Wnt pathway components (Wnt1, Wnt8b) and cell-cycle genes (AURKA, AURKB), depicted in red to indicate inhibition. In contrast, SIX3 also functions as a transcriptional activator of neural/ocular identity genes, such as Pax6 and Sox2 (shown in green to indicate activation), promoting neural progenitor specification and eye-field formation. (**B**) Temporal and spatial roles of SIX3 and SIX6 in neurogenesis. SIX3 is expressed early in highly proliferative neuroepithelial cells, where it preserves neuroepithelial architecture, prevents premature differentiation, and maintains developmental plasticity, in part through regulation of Foxg1, Rx3/Rax, and cell-cycle regulators. As development proceeds, SIX6 expression increases in later progenitor populations of the retina, optic stalk, and hypothalamus, sustaining late progenitor pools, supporting neuronal survival, and coordinating the balance between proliferation and differentiation within domains where SIX3 and SIX6 activities overlap.

SIX3 binds specific regulatory elements of these genes and enhances their transcription. Thus, mutations in SIX3’s activation or repression domains proportionally alter reporter activity, confirming that its transcriptional activity depends on the integrity of its functional domains [24] (Figure 2A).

Although classical activating partners such as Eya are well established for other SIX family members (e.g., SIX1 and SIX2), for SIX3 this interaction has not been demonstrated in vertebrates [19]. However, in *Drosophila*, the SIX3 ortholog *optix* and *eya* are both direct transcriptional targets of the retinal determination factor Eyeless, indicating a functional regulatory connection between SIX3/Optix and Eya in the retinal determination gene network, even if a direct protein–protein interaction is not observed [25].

Mechanistically, SIX3 repressive activity is largely mediated by interactions with Groucho/TLE family corepressors. Protein interaction studies have shown that the SIX domains of SIX3 and SIX6 bind strongly to TLE1 and the antagonist AES [22,38,39] (Table 1) (Figure 2A). In addition to these interactions via the SIX domain, SIX3 can additionally engage WDR domains of Groucho cofactors, suggesting multiple modes of interaction that modulate repressive activity depending on the context and cofactor composition [22,40]. The SIX3/SIX6 proteins not only interact with their regulators through their SIX domains but also through their disordered regions. Despite low sequence conservation, C- and N-terminal disordered regions contribute to interactions with regulatory partners such as GEMININ [35,41].

These shared interaction properties highlight the structural and mechanistic relatedness of SIX3 and SIX6, but accumulating evidence indicates that the two paralogs are deployed in partially distinct developmental and disease contexts. SIX6 was first characterized as a distinct SIX3-related homeobox gene with specific expression in the retina, hypothalamus, and pituitary, indicating a developmental role separable from SIX3 [42]. Functional studies in mouse models show that loss of SIX6 in GnRH neurons leads to decreased GnRH expression and infertility, underscoring a non-redundant role in hypothalamic/neuroendocrine development distinct from SIX3 [26]. Furthermore, SIX6 regulates the transcription of pituitary-specific genes via TLE cofactors, differing in timing and targets from SIX3 [42]. In the retina, conditional and compound knockout studies reveal that SIX6 contributes to progenitor maintenance and differentiation, with phenotypes not fully recapitulated by loss of SIX3 alone [27]. These differences support the view that, despite shared structural modules and cofactors, SIX3 and SIX6 are deployed in distinct temporal and tissue-specific regulatory programs.

Reflecting differences in developmental roles, the roles of SIX3 and SIX6 in cancer are substantially different. SIX6 has been found to be mostly associated as a poor prognosis marker of different cancer types but where the causality or the molecular role is still to be determined [43,44,45]. In contrast, SIX3 is mostly associated with tumour suppression, and the regulatory mechanisms are better characterized. In transformed cell lines, SIX3 directly interacts, via non-canonical regions near the homeodomain, with LSD1, and through the SIX domain and adjacent sequences mediates interactions with MTA3, a component of the NuRD chromatin-remodelling complex (Table 1) [42]. These interactions enable SIX3 to recruit the NuRD complex to promoter regions and, through modulation of nucleosome occupancy, coordinately regulate multiple components of signalling pathways involved in cell growth, survival, migration, and invasion [42]. Whether similar mechanisms operate during development, where SIX3 activity must be precisely fine-tuned, and how robust or reversible such epigenetic regulation is within a developing organism remain important open questions.

## 3. Developmental Roles of SIX3 in Cell Fate Specification

The SIX3/SIX6 subfamily plays two major roles across metazoans, a crucial role during development, where it regulates anterior neural and eye formation, and a role in maintaining homeostatic balance in differentiated tissues. As *Drosophila* only presents a single member of this family, it is likely that it might fulfil all the roles of both SIX3 and SIX6. Pioneer studies of the developmental roles of Optix came from studies in the visual system of *Drosophila*. The functional ortholog Optix is expressed in defined domains of the eye imaginal disc, particularly anterior to the morphogenetic furrow, where it stabilizes cell identity, regulates proliferation, and prevents activation of alternative fate programs [25,46]. Beyond its role within the retinal determination network, Optix also participates in the spatial organization of morphogenetic signalling [46]. Functional analyses demonstrated that Optix is required for proper Dpp signalling dynamics during eye development, acting to coordinate the progression and spatial restriction of the morphogenetic furrow rather than initial eye field specification [46]. Importantly, Optix function extends beyond the eye imaginal disc and into neuroepithelial contexts.

In the developing *Drosophila* brain, Optix is expressed in discrete neuroepithelial compartments of the optic lobe, regions that give rise to medulla and lamina neurons, key elements of the visual system, where it plays a critical role in maintaining compartment boundaries and epithelial integrity. Loss of Optix in this context results in mixing of adjacent neuroepithelial domains, altered cell adhesion properties, and defects in compartmental organization, without directly altering neuronal fate specification [47]. Furthermore, other patterning factors such as Spalt and Disco also define dorsal–ventral axes of the optic lobe neuroepithelium, revealing that multiple transcriptional inputs cooperate with Optix to organize spatial domains and diversify neural progenitors within the visual system [48].

Furthermore, Optix also plays a role in the formation of other epithelial tissues. Optix is expressed in the distal-anterior region of the wing pouch of the wing imaginal disc, where it delineates domains of growth, patterning, and vein formation [49]. Loss of Optix in this tissue results in defective wing expansion and margin formation, highlighting a conserved role in progenitor maintenance and identity regulation [49,50]. Optix is also expressed in the haltere imaginal disc, in central domains that contribute to growth regulation and sensilla formation [50].

These molecular principles are conserved across metazoans. In vertebrate model organisms, including mouse, zebrafish, and Xenopus, the transcription factors SIX3 and SIX6 act in a coordinated but non-redundant manner, regulating distinct phases of anterior neural fate establishment, maintenance, and differentiation. SIX3 also directly represses key Wnt pathway genes, including *wnt1* and, in some contexts, *wnt8b*, through binding to regulatory elements during anterior neural development [23,36,51]. This function is conserved across species, in SIX3^−/−^^(−/−)^mouse models, Wnt1 expression expands rostrally, disrupting normal head development [51], whereas ectopic expression of SIX3 in zebrafish embryos represses Wnt1 and can rescue “headless” phenotypes [23] (Figure 2A). In contrast, SIX6 plays a prominent role in the maintenance and proliferation of retinal progenitors in this developmental process [52]. Similarly, in Xenopus, SIX3 and SIX6 cooperate during anterior neural plate specification but exhibit only partial functional overlap [53], and in zebrafish SIX3 shows a specific requirement for forebrain and eye development through transcriptional regulation of downstream targets, such as *wnt1* and *wnt8b* [37]. Additionally, overexpression of XOptx2 in Xenopus leads to enlarged eye structures, highlighting the role of Optix in eye field specification and growth [54].

The anterior neuroepithelium represents a highly sensitive domain in which signalling gradients and transcriptional regulatory mechanisms converge to establish the anterior–posterior axis. In this context, SIX3 is expressed early in the anterior neural plate, at a stage when neuroepithelial cells still exhibit high developmental plasticity [23]. Its activity during this initial period is critical for stabilizing anterior identity and preventing the premature activation of differentiation or posterior identity programs [23,55]. Loss-of-function studies in murine models demonstrate that SIX3 deficiency leads to rostral expansion of posteriorizing signalling domains, disorganization of neuroepithelial architecture, reduced proliferation of neural progenitors, and severe forebrain malformations, indicating that SIX3 functions as a guardian of early neuroepithelial competence [56]. Beyond its role in maintaining regional identity, SIX3 cooperates with other anterior determinants, including Foxg1 and Rx3/Rax, to reinforce anterior fate specification and constrain caudalizing signals, thereby establishing precise spatial boundaries within the forebrain and eye field [36] (Figure 2B). Furthermore, SIX3 regulates progenitor behaviour by modulating the expression of genes involved in cell cycle progression, such as Cyclin D1 and p27Kip1 (Table 2) and neuroepithelial architecture, promoting proliferation and maintaining precursor cells in an undifferentiated state [55,56]. Through its dual role as a transcriptional activator and repressor, SIX3 integrates positional cues while maintaining the progenitor pool and ensuring proper regional patterning [31].

In zebrafish, reduced function of SIX3 results in optic nerve hypoplasia, severe defasciculation of retinal ganglion cell (RGC) axons, and pathfinding errors during projection across the retina and diencephalon, phenotypes that mirror aspects of human congenital eye malformations [57]. These defects arise from multiple developmental mechanisms, including abnormal patterning of the eye and optic stalk, disrupted expression of guidance receptors and ligands, and deficiencies in the diencephalic preoptic area where the optic chiasm forms, indicating that SIX3 is required for normal RGC development and optic nerve morphogenesis [57].

In contrast, SIX6 expression initiates later and becomes progressively restricted to specific subdomains of the neuroepithelium, including the developing retina, optic stalk [58], and hypothalamic regions [59]. Rather than participating in the initial anterior specification of the neuroepithelium, SIX6 functions predominantly at subsequent developmental stages, where it promotes the expansion, survival, and differentiation of neuronal populations that have already acquired regional identity [59,60]. Functional studies in vertebrate models demonstrate that SIX6 enhances the proliferative capacity of neural and retinal progenitors [60], supports the maintenance of precursor pools during late neurogenesis [59], and is required for the survival of specific neuronal lineages, including hypothalamic GnRH neurons, whose loss in SIX6-deficient models results in increased apoptosis and severe developmental defects [60].

Despite these temporal differences, SIX3 and SIX6 display partially overlapping expression domains within the anterior neuroepithelium and the developing retina [58,61]. These co-expression domains represent potential zones of functional cooperation, in which SIX6 can partially compensate for the loss of SIX3 at later developmental stages, but not during early anterior patterning [61]. This functional hierarchy suggests that SIX3 occupies a dominant position within the regulatory network governing anterior neuroepithelial identity, whereas SIX6 acts primarily as a modulator of tissue expansion and differentiation [52].

Coordination between SIX3 and SIX6 is also evident during ocular field formation. SIX3 is indispensable for establishing the initial eye territory within the anterior neuroepithelium, thereby conferring competence for optic vesicle formation. Subsequently, SIX6 contributes to neural retina expansion and regulates the proliferation of retinal precursor cells [58,61,62]. Disruption of this balance results in distinct ocular phenotypes, ranging from early eye field defects following SIX3 loss to impaired retinal growth and differentiation observed in SIX6-deficient models [63].

Putting together, these studies using model organisms identified the commonalities of the SIX3/SIX6 subfamily during development across different species, principles that have a severe impact on human development and underlie many pathological conditions that will be explored in the next sections.

## 4. SIX3 and SIX6 in Neurodevelopmental and Degenerative Eye Diseases

Beyond their essential roles in embryogenesis, alterations in the expression or function of SIX3/SIX6 subfamily are strongly associated with a broad spectrum of neuro-ocular pathologies [4,31].

In humans, heterozygous mutations in SIX3 are a well-established genetic cause of holoprosencephaly (HPE), a severe congenital brain malformation characterized by incomplete separation of the forebrain into bilateral cerebral hemispheres. Depending on the nature and location of the variant, phenotypes range from classic HPE to more severe forms such as aprosencephaly and atelencephaly, often accompanied by craniofacial anomalies and ocular defects including cyclopia, anophthalmia or microphthalmia [64,65]. In HPE, pathogenic variants in SIX3 disrupt its ability to bind DNA and regulate downstream targets that are critical for forebrain and eye field patterning, such as repression of posteriorizing Wnt signals and activation of Sonic Hedgehog (SHH) and PAX6 during early development [24,66]. Specific missense mutations in functional domains, such as V250A and R257P in the homeodomain that impair DNA binding, and V92G and H173P in the SIX domain affecting protein interactions, have been described in HPE patients, highlighting the importance of these regions for proper transcriptional control [66].

These genotype-phenotype correlations are supported by functional assays in zebrafish and other model systems, which show that many disease-associated SIX3 mutations result in a significant loss of transcriptional function during early embryonic stages when the anterior neural plate is being specified. In zebrafish, this corresponds to approximately 6 to 12 h post fertilization, a period critical for forebrain and eye field development [64], while in mice, the equivalent window occurs around embryonic day 7.5 to 8.5, when ventral forebrain patterning and Shh expression are initiated [66].

In addition to congenital malformations associated with SIX3, genetic variants at the SIX6 locus have been consistently implicated in primary open-angle glaucoma (POAG), a common neurodegenerative optic neuropathy and leading cause of irreversible blindness worldwide. Genome-wide association studies (GWAS) have identified signals at the SIX1–SIX6 locus that are significantly associated with structural parameters of the optic nerve, including the vertical cup-to-disc ratio, and with increased POAG risk in multiple populations [67,68,69]. Functional exome sequencing and follow-up in vivo assays in zebrafish demonstrated that several SIX6 missense variants, including the common rs33912345 (Asn141His) allele, are hypomorphic or loss-of-function and confer altered eye size and optic nerve structure. Moreover, individuals homozygous for the risk allele exhibit thinner retinal nerve fiber layers, consistent with reduced retinal ganglion cell numbers and enhanced susceptibility to glaucomatous degeneration [70,71].

## 5. Context-Dependent Roles of SIX3 and SIX6 in Tumourigenesis

Despite many studies focusing on the roles of SIX3 and SIX6 in normal development, increasing evidence implicates these transcription factors in tumourigenesis and cancer progression across multiple histological types. Aberrant expression of SIX family genes has been observed in tumour specimens and cell lines, frequently associated with increased proliferation, invasion, metastasis, and poor clinical outcomes, reflecting their context-dependent roles as oncogenes or tumour suppressors.

While SIX6 is generally associated with pro-tumourigenic functions, promoting proliferation, invasion, and stem-like properties [43,44,45], SIX3 can display context-dependent oncogenic activity.

In gliomas and astrocytomas, SIX3 has been characterized as a tumour suppressor. SIX3 expression is frequently downregulated in these malignancies through promoter hypermethylation, and its restoration inhibits malignant phenotypes by repressing key mitotic regulators. Chromatin immunoprecipitation and luciferase reporter assays demonstrated that SIX3 directly binds to and represses transcription of *aurora kinase A* (AURKA) and *aurora kinase B* (AURKB), two kinases critical for centrosome maturation, chromosome segregation, and mitotic progression [72]. Overexpression of SIX3 decreases AURKA and AURKB expression, increases p53 activity at a post-translational level and reduces numerical and structural centrosomal aberrations. This regulation significantly inhibits proliferation, invasion, and tumour formation in vitro and in vivo in astrocytoma models, supporting a tumour suppressive role [72].

Mechanistically, the SIX3 tumour suppressor activity is subjected to epigenetic regulation. Activation of oncogenic pathways, including EGFR-MAPK, leads to hypermethylation and transcriptional silencing of the SIX3 promoter through recruitment of ZNF263 and associated corepressor complexes [73]. This results in reduced SIX3 expression and enhanced tumourigenic activity, indicating that SIX3 promoter hypermethylation may act as a driving factor in tumour initiation and progression in glioblastoma [73]. This notion is further supported by studies in lung adenocarcinoma showing a correlation between SIX3 promoter hypermethylation and poor prognosis [74], as well as by functional assays in glioma models in which reversal of promoter methylation restored SIX3 expression and reduced proliferation [75].

In addition to its role in controlling mitotic regulators, SIX3 also exerts tumour-suppressive functions through repression of oncogenic developmental signalling pathways. SIX3 directly represses Wnt1 by binding to its regulatory elements and limiting Wnt/β-catenin pathway activation [42]. Loss of SIX3, whether through promoter hypermethylation or depletion, leads to Wnt1 derepression, aberrant activation of Wnt/β-catenin signalling, and enhanced tumour cell proliferation and invasion [42,75]. In breast cancer models, SIX3, together with the NuRD complex, represses WNT1 transcription, and SIX3 depletion increases WNT1 expression and proliferation effects partially reversed by WNT1 knockdown [42]. Similarly, in glioma cells, epigenetic silencing of SIX3 correlates with elevated Wnt1 levels and downstream β-catenin signalling, further promoting tumour progression [75]. These findings indicate that SIX3 loss cooperates with oncogenic drivers not only by disrupting cell cycle control but also by unleashing developmental signalling pathways that contribute to malignancy.

Although SIX3 is predominantly described as a tumour suppressor in many cancer types, evidence from specific contexts suggests that modulation of SIX3 activity can contribute to oncogenic signalling and tumour progression rather than inhibition. In esophageal cancer, upregulation of SIX3 has been associated with increased proliferation, invasion, and migration, as well as poor clinical prognosis, supporting a context-dependent pro-tumorigenic role in which elevated SIX3 expression correlates with aggressive tumour behaviour [76]. Additionally, clinical analyses of the SIX family in NSCLC have reported paradoxical patterns of SIX3 expression, with higher SIX3 mRNA levels detected in tumour tissues correlated with advanced tumour stage in certain NSCLC subgroups, suggesting that SIX3 may promote tumour initiation or progression at specific stages or in particular cellular contexts [77]. These findings underscore that, while loss of SIX3 frequently accompanies tumour suppressive loss-of-function, its regulatory network can be exploited by oncogenic drivers to enhance malignancy in a context-dependent manner.

Further studies investigating how the expression of the SIX3/SIX6 family is regulated, what their interaction partners are in each cellular state, and what targets they regulate in different contexts will be essential for understanding how to modulate these proteins and may provide important clues for the development of improved future therapies.

## 6. Cooperative Roles of SIX3 and SIX2 in Pancreatic β-Cell Maturation and Type 2 Diabetes

Beyond its established roles in multiple vertebrate developmental contexts as a master regulator of cellular identity programs, SIX3 has recently been implicated in the functional maturation of human pancreatic β-cells, highlighting conserved mechanisms of cell fate regulation across distinct tissues [78]. Notably, these functions have been demonstrated directly in primary human islets and pseudo-islet systems, and are not fully recapitulated in murine β-cells, where SIX2 and SIX3 expression is minimal or absent, underscoring the existence of a predominantly human-specific maturation axis [78,79]. During postnatal β-cell development, SIX3 and its paralog SIX2 are coordinately upregulated, coinciding with the acquisition of key functional competencies such as glucose-stimulated insulin secretion (GSIS) [78,79].

**Figure 3:**
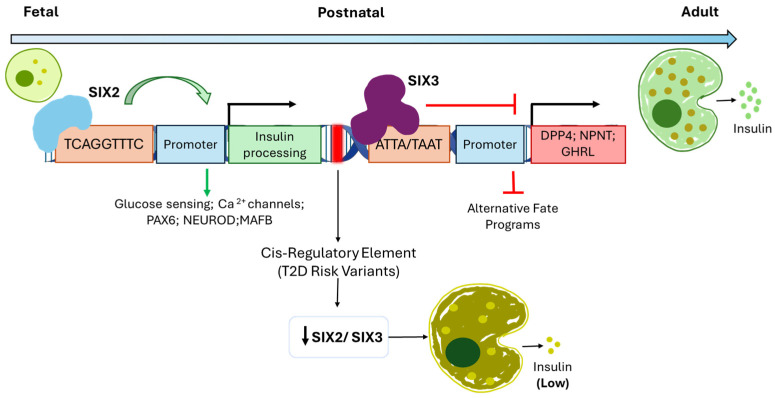
SIX2/SIX3 regulatory network in human β-cell maturation and dysfunction. Schematic representation of how a shared cis-regulatory element controls the coordinated postnatal induction of SIX2 and SIX3 in human β-cells, promoting functional maturation programs (insulin processing, glucose sensing, Ca^2+^ signalling, and key transcription factors) while repressing immature or alternative fate genes such as DPP4, NPNT, and GHRL to stabilize β-cell identity. In type 2 diabetes, reduced SIX2/SIX3 expression is proposed to weaken these maturation networks and relieve repression of alternative gene programs, contributing to β-cell dedifferentiation, impaired insulin secretion, and increased disease susceptibility.

Mechanistically, SIX2 acts as a direct activator of gene programs underlying adult β-cell maturation. Knockdown of SIX2 in human pseudo-islets significantly impairs GSIS without affecting total insulin content, indicating a specific requirement for functional competence rather than insulin production per se [78]. Importantly, these functional effects were established using human donor islets, providing direct evidence for SIX2-dependent maturation mechanisms in the human β-cell context [78]. Transcriptomic and chromatin accessibility analyses demonstrate that SIX2 directly regulates genes involved in insulin processing and secretion, glucose sensing, electrophysiological signalling, and key maturation factors, including PAX6, NEUROD1, and MAFB, as well as genes mediating glucose–insulin coupling [78]. In contrast, SIX3 primarily represses alternative or immature gene programs. Loss of SIX3 in adult β-cells leads to the aberrant expression of α-cell– or foetal-associated genes such as DPP4, NPNT, and GHRL (Table 2), supporting a role for SIX3 in stabilizing mature β-cell identity by suppressing incompatible transcriptional states [78]. Consistently, overexpression of SIX3 in immature human islets enhances glucose-dependent insulin secretion, reinforcing its contribution to β-cell functional maturation alongside SIX2 [78,79] (Figure 3).

Importantly, the coordinated and balanced activity of SIX2 and SIX3 constitutes a transcriptional network that safeguards both β-cell identity and secretory competence throughout adult life. Their expression is controlled by a shared cis-regulatory element within pancreatic islets that harbours genetic variants associated with fasting glycemia and increased risk of type 2 diabetes (T2D) in genome-wide association studies [80]. This genetic link directly implicates the SIX2/SIX3 regulatory module in β-cell susceptibility to metabolic disease (Figure 3).

Strikingly, expression of both SIX2 and SIX3 is significantly reduced in β-cells from individuals with T2D, particularly in cases characterized by impaired insulin secretion [78]. This reduction is consistent with a model in which disruption of the SIX2/SIX3 transcriptional network contributes to β-cell dedifferentiation, loss of functional maturity, and failure to appropriately respond to metabolic demand. In this context, diminished SIX2 activity may compromise insulin secretion by weakening core maturation gene programs, while reduced SIX3 expression may permit the reactivation of immature or non–β-cell gene expression, exacerbating β-cell dysfunction.

These observations position SIX2 and SIX3 not merely as markers of β-cell maturity, but as active regulators whose dysregulation may drive β-cell failure in T2D. Supporting this notion, studies in stem cell–derived β-like cells demonstrate that induction of SIX2 and SIX3 expression correlates with enhanced glucose responsiveness and maturation-associated transcriptional signatures, reinforcing their potential as therapeutic targets for β-cell replacement or regeneration strategies [81,82]. Beyond their value as maturation markers, these findings raise the possibility that targeted modulation of SIX2/SIX3 activity could be leveraged to enhance functional maturation of stem cell–derived β-like cells prior to transplantation, improve durability of β-cell identity under metabolic stress, or counteract dedifferentiation processes observed in T2D. Such approaches may complement current β-cell replacement and regenerative strategies by focusing not only on cell number but also on transcriptional state stability [78,82]. Collectively, these findings suggest that restoring or stabilizing the SIX2/SIX3 regulatory axis could represent a promising avenue to preserve β-cell identity and function in diabetes, with implications for disease modelling, human β-cell engineering, and the development of transcriptionally guided therapeutic interventions.

## 7. Discussion and Future Directions

Given their fundamental roles in organismal development and tissue homeostasis, SIX proteins are subject to tight regulatory control. As discussed above, their regulation occurs at multiple levels, ranging from transcriptional control to differential interaction with co-factors. While several studies have characterized regulatory mechanisms operating during development, how SIX proteins, particularly SIX3, are regulated during adult homeostasis remains largely unexplored.

In this review, we focused on the SIX3/SIX6 subfamily because of the unique properties of SIX3. Whereas other SIX subfamilies recognize DNA motifs TCAGGTTTC, SIX3/SIX6 preferentially binds the ATTA/TAAT motif. Given the high prevalence of this sequence associated with multiple promoters in the genome, it is somewhat surprising that SIX3 has been described primarily as a transcriptional repressor of a few key developmental regulators. This observation suggests that SIX3 activity must be tightly constrained in a context-dependent manner, both spatially and temporally, to ensure selective target engagement.

One important mechanism controlling SIX3 activity is transcriptional regulation by upstream developmental factors, as illustrated by its regulation by the PAX6/Ey transcription factor [25]. In addition, SIX3 participates in negative feedback loops that restrict its own transcription [29], contributing to the fine-tuning of its expression during development. However, how these regulatory mechanisms are adapted to achieve precise target selection during timing-sensitive differentiation events and in adult tissues remains unclear. Identifying the direct protein partners of SIX3 will therefore be essential to understanding how its repressive activity is selectively directed toward specific genomic loci.

To complement protein–protein interaction studies, systematic mapping of SIX3 chromatin occupancy by ChIP-seq across developmental stages will be critical for defining its direct transcriptional targets and regulatory logic. Such approaches may help resolve the paradoxical behaviour of SIX3 in cancer, where it functions as a tumour suppressor in some tumour types yet correlates with poor prognosis in others. These contrasting roles strongly suggest that context-specific cofactors, chromatin environments, or upstream regulatory inputs modulate SIX3 DNA binding and target gene selection.

Further investigation of SIX3 function during both development and adult homeostasis will be necessary to define the molecular players that regulate its activity and shape its transcriptional output. In this regard, epigenetic mechanisms are likely to play an important role. On the one hand, the SIX3 promoter is frequently hypermethylated in several cancer types [72], indicating that epigenetic silencing may contribute to altered SIX3 expression in disease. On the other hand, in transformed cell lines, SIX3 has been reported to interact with the NuRD chromatin remodelling complex, and actively recruiting repressive chromatin machinery to genes involved in cell growth, survival, migration and invasion [42].

This raises the possibility of a two-step regulatory model in which SIX3 might initially repress transcription through direct DNA binding and might subsequently promote stable chromatin remodelling via NuRD-mediated nucleosome reorganization, thereby locking cells into a differentiated transcriptional state and preventing reactivation of alternative lineage programs. Determining whether SIX3-dependent recruitment of NuRD alters chromatin accessibility at proliferation- or plasticity-associated genes during development or homeostatic conditions and the reversibility of this mechanism will be particularly important for understanding the potential of SIX3 as a therapeutic target in cancer.

Notably, many of the epigenetic mechanisms implicated in SIX3 dysregulation in cancer, such as promoter hypermethylation and chromatin remodelling, are increasingly recognized as central regulators of β-cell identity, plasticity, and failure in metabolic disease.

In human pancreatic β-cells, SIX3 has been shown to contribute to the maintenance of mature functional identity, and reduced expression of SIX3 has been observed in β-cells from individuals with impaired insulin secretion and type 2 diabetes [78]. These observations establish a correlative link between SIX3 dysregulation and β-cell dysfunction but do not yet resolve whether altered SIX3 activity represents a causal driver of disease or a secondary consequence of metabolic stress. Although epigenetic silencing of the SIX3 locus through promoter hypermethylation and recruitment of chromatin remodelling complexes such as NuRD have been described in other biological contexts, it remains unknown whether similar mechanisms operate in diabetic β-cells. It will therefore be important to determine whether changes in chromatin accessibility, DNA methylation, or cofactor availability influence SIX3 expression or target gene selection during β-cell failure. Addressing these questions may clarify whether impaired SIX3 function contributes to β-cell dedifferentiation, loss of functional maturity, or reduced secretory capacity in diabetes. Ultimately, defining the regulatory mechanisms governing SIX3 in healthy and diseased β-cells could inform the development of strategies aimed at preserving or restoring β-cell identity and function, as well as the identification of biomarkers reflecting β-cell maturity and disease progression.

## 8. Conclusions

In conclusion, accumulating evidence positions SIX3 as a pivotal and highly context-dependent transcriptional regulator whose activity is shaped by multilayered control mechanisms. Although its essential functions during early development are well established, its regulation and roles in adult tissue homeostasis remain incompletely defined. The apparent paradox between the widespread occurrence of its binding motif and its seemingly restricted set of described targets underscores the importance of spatial, temporal, and chromatin-based constraints that refine its genomic engagement.

Emerging data support a model in which SIX3 activity is governed not only by upstream transcriptional networks and feedback loops, but also by dynamic interactions with chromatin-modifying complexes and epigenetic mechanisms that stabilize transcriptional outcomes. Context-specific cofactors and chromatin landscapes likely determine whether SIX3 acts as a tumour suppressor, a context-dependent oncogenic factor, or a guardian of differentiated cell identity. Resolving these context-dependent functions will require integrative approaches combining chromatin profiling, protein–protein interaction mapping, and functional studies across developmental and adult tissues. Those approaches will not only clarify the fundamental biology of SIX3 but may also open new avenues for therapeutic strategies aimed at preserving cell identity, preventing dedifferentiation, and modulating disease progression in cancer and metabolic disorders.

## Figures and Tables

**Table 1:** Multilayered regulatory mechanisms controlling SIX3 expression and activity.

		Mechanism	Context	Effect on SIX3
Transcriptional	PAX6/Ey	Direct promoter/enhancer activation	Eye and neural development	Activates SIX3 transcription
	Msx2, Pbx1, Tcf3	Direct promoter/enhancer activation	Forebrain specification	Spatial Control of SIX3 transcription
	MTA1	NuRD complex recruitment; HDAC1/HDAC2-mediated histone deacetylation	Cancer (glioblastoma, NSCLC, esophageal cancer)	Represses SIX3 transcription
	Promoter hypermethylation	DNA methylation-mediated silencing	Cancer (Glioma, lung cancer)	Silencing of SIX3
RNA-mediated Regulation	miR-4306	3′UTR targeting; mRNA destabilization	Esophageal carcinoma	Downregulates SIX3 mRNA
	Six3OS1 (lncRNA)	RNA–protein interaction; chromatin modulation	Retinal development	Modulates SIX3 levels
Protein Interaction	TLE1/AES	Corepressor recruitment	Development and cancer	Enables repression function
	MTA3 (NuRD component)	SIX3 recruits MTA3-containing NuRD complex; chromatin remodeling	Development and cancer	Transcriptional repression of SIX3 target genes
	LSD1	SIX3 recruits LSD1 histone demethylase; chromatin remodeling	Development and cancer	Transcriptional repression of SIX3 target genes

**Table 2:** Representative SIX3 target genes across developmental and disease contexts.

		Target	Biological Context
Development	Activation by SIX3	Pax6	Neural and ocular identity
		Sox2	Neural progenitor specification
		Foxg1	Forebrain specification
		Rhodopsin	Retinal specification
		Cyclin D1	Neural progenitor proliferation and cell-cycle progression
	Repression by SIX3	SIX3 (self-promoter)	Neural/eye specification
		p27Kip1	Control of neural progenitor cell-cycle exit
		Wnt8b	Forebrain/eye field patterning
		DPP4, NPNT, GHRL	Stabilization of mature β-cell identity and repression of immature gene programs
		Wnt1	Anterior neural specification
Adult		AURKB	Repression of cell cycle progression in tumourigenesis
		AURKA	Repression of cell cycle progression in tumourigenesis
		Wnt1	Oncogenic Wnt signalling

## Data Availability

No new data were created or analyzed in this study. Data sharing is not applicable to this article.
